# Taurine: the appeal of a safe amino acid for skeletal muscle disorders

**DOI:** 10.1186/s12967-015-0610-1

**Published:** 2015-07-25

**Authors:** Annamaria De Luca, Sabata Pierno, Diana Conte Camerino

**Affiliations:** Sezione di Farmacologia, Dipartimento di Farmacia-Scienze del Farmaco, Università degli Studi di Bari “Aldo Moro”, Bari, Italy

**Keywords:** Taurine skeletal muscle, Inherited muscle disorders, Disuse muscle atrophy, Development and aging, Skeletal muscle performance

## Abstract

Taurine is a natural amino acid present as free form in many mammalian tissues and in particular in skeletal muscle. Taurine exerts many physiological functions, including membrane stabilization, osmoregulation and cytoprotective effects, antioxidant and anti-inflammatory actions as well as modulation of intracellular calcium concentration and ion channel function. In addition taurine may control muscle metabolism and gene expression, through yet unclear mechanisms. This review summarizes the effects of taurine on specific muscle targets and pathways as well as its therapeutic potential to restore skeletal muscle function and performance in various pathological conditions. Evidences support the link between alteration of intracellular taurine level in skeletal muscle and different pathophysiological conditions, such as disuse-induced muscle atrophy, muscular dystrophy and/or senescence, reinforcing the interest towards its exogenous supplementation. In addition, taurine treatment can be beneficial to reduce sarcolemmal hyper-excitability in myotonia-related syndromes. Although further studies are necessary to fill the gaps between animals and humans, the benefit of the amino acid appears to be due to its multiple actions on cellular functions while toxicity seems relatively low. Human clinical trials using taurine in various pathologies such as diabetes, cardiovascular and neurological disorders have been performed and may represent a guide-line for designing specific studies in patients of neuromuscular diseases.

## Background

**Fig. 1:**
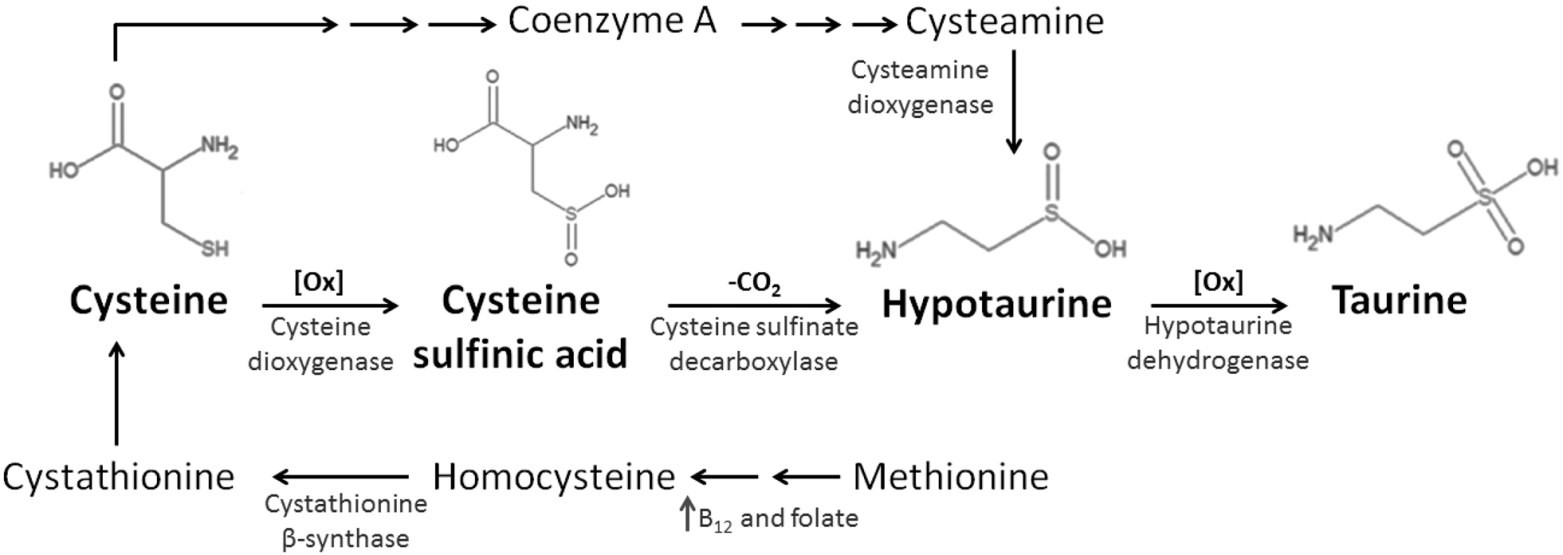
Biosynthetic route of taurine from amino acids cystein and methionine. The synthesis does primarily occur in liver, although other tissues can contribute to its synthesis based on presence of key enzymes. However, the tissue synthesis is generally low, and tissues needs to uptake circulating taurine against gradients by means of the specific Na^+^/Cl^−^ dependent transport system, TauT. Some species (i.e. felines) cannot synthesize taurine and dramatically depends on taurine intake with food. Diet is indeed an important source of the amino acid for all species, especially if reach of fish or beef meat as well as other animal-derived food (i.e. milk).

Taurine (2-aminoethane-sulfonic acid) is a sulfur-containing amino acid which is not used for protein synthesis and is therefore the most abundant free amino acid in mammalian tissues, with the exception of human liver in which aspartate is the most abundant one [1, 2]. The intracellular concentration of taurine ranges between 5 and 20 µmol/g wet weight in many tissues, especially in excitable ones, such as brain, heart and skeletal muscle [1, 3, 4]. Endogenous synthesis occurs in the liver via the cysteine sulfinic acid pathway. The metabolic reaction consists in a first oxidation of the sulfhydryl group of cysteine to cysteine sulfinic acid by the enzyme cysteine dioxygenase. Cysteine sulfinic acid is then decarboxylated to hypotaurine by the cystyeine sulfinate decarboxylase. Taurine is obtained by a yet unclear spontaneous or enzymatic oxidation (by hypotaurine dehydrogenase) of hypotaurine (Fig. 1). The endogenous synthesis of taurine is highly variable between individuals also in relation to nutritional state, to the amount of protein intake and to cysteine availability [1, 5]. In turn the availability of cysteine is highly dependent on the metabolic equilibrium between homocysteine and methionine, via folic acid, vitamin B12 and the efficiency of the enzyme methyltetrahydrofolate reductase. In addition, a certain amount of taurine has to be introduced with food, mostly in carnivores and, to a minor extent, in omnivores [1]. The importance of the two sources vary quite a lot between species, with some, like felines and foxes, being highly dependent on diet acquisition of taurine, as they are unable to synthesize it. These species are also particularly susceptible to deficient states, developing severe pathophysiological conditions, such as dilated cardiomyopathy, retinal degeneration and reproduction defects [3, 6]. These evidences first outlined the key role of taurine for mammalian tissue functions and helped to better understand the link between tissue distress in retaining proper taurine concentration and various pathophysiological conditions.

In fact, even in species able to synthesize taurine, the tissue-specific synthesis is relatively low, with liver being the main source according to the higher expression of enzymes as cysteine dioxygenase. Importantly, the activity of this latter enzyme strictly depends upon cysteine availability, so that the exact amount of taurine being endogenously synthesized is difficult to predict [7]. However, the high intracellular concentration is guaranteed by the presence of a specific active transporter that concentrates taurine inside the cells against gradients. The taurine transporter (TauT; encoded by the SLC6A6 gene) is a sodium and chloride ion-dependent transporter ubiquitously expressed in mammalian tissues. The concentration of taurine is 100-fold less in the plasma (20–100 µM) than in the tissues, suggesting that it is indeed required for modulating key cellular functions. Due to the high tissue concentration, taurine also works as an osmolyte. Its cellular efflux via volume-dependent or volume-independent pathways works to osmotically balance the excessive production of metabolic by-products. Both uptake systems and efflux pathways are tightly regulated at transcriptional and post-transcriptional level, leading to an accurate control of taurine intracellular levels [8].

Since its discovery in ox bile in 1827, several physiological functions have been described for the amino acid, ranging from the classical role of conjugating agent for bile acids, to wider actions as osmotic pressure regulator, modulator of calcium homeostasis and signaling and, more recently, as an endogenous anti-oxidant and anti-inflammatory compound in various tissues. The mechanism by which taurine exerts all these different functions is still unclear. Some of the taurine actions in central nervous system (CNS), seem to occur via specific binding sites or receptors, i.e. in thalamus taurine modulates neuronal firing via activation of extra-synaptic gamma-amino butyric acid (GABA) receptor isoforms α4β2δ with a greater affinity than GABA [9–12]. Such high affinity binding sites have not been evidenced in other tissues.

Skeletal muscle is one of the tissues able to concentrate the largest amount of body’s taurine, via the TauT activity. Pioneer studies of Ryan Huxtable anticipated that the high taurine level is needed to maintain an appropriate calcium homeostasis, likely by ensuring a correct calcium re-uptake by the sarcoplasmic reticulum [13]. Similar actions were also described in heart, with taurine exerting complex modulation of calcium homeostasis in relation to external concentration of the cation with beneficial effects in contrasting arrhythmias or heart failure [1, 3, 4].

Transgenic mice lacking TauT gene have been generated by two separate groups [6, 14–16]. In line with a key role of taurine for maintaining proper physiological functions, the drastic reduction in content consequent to TauT deletion is associated to a variety of disorders in various tissues, such as eye, kidney, heart, nociceptive system and skeletal muscle [14–17]. These conditions resemble those occurring when taurine tissue content is altered by pathophysiological states or by inhibitors of the taurine transporter. In spite the pre-clinical research has disclosed many conditions in which taurine supplementation may be beneficial, the therapeutic use of taurine is very limited. Taurine is commonly known for its claimed effects as energizer and anti-fatigue compound and it is present in many energy soft drinks as well as in supplement cocktails for athletes. The toxicity of taurine in this context is considered relatively low with respect to other active ingredients; actually it may also be protective against cardiovascular action of caffeine [18]. Such a protection may again result from multiple taurine actions, i.e. an antihypertensive effect via vasodilatation (by reducing adrenergic and angiotensin II actions as well as calcium-induced vasospasm) along with a reduced risk of cardiac arrhythmias via modulation of ion channels and ionic homeostasis [18]. However a certain caution is important especially when taurine is used in children and/or in association with drugs, alchool or other food supplements [19–23]. Apart for its nutraceutical role, taurine may exert clear pharmacological actions by modulating signaling pathways and targets or via restoration of its altered tissue levels. No systematic toxicity studies have been performed to assess the toxicological parameters for taurine; however human trials have used taurine up to 10 g/daily without overt signs of toxicity. This may also depend on the direct relationship between taurine plasma level and its excretion rate by the kidney [19].

**Fig. 2:**
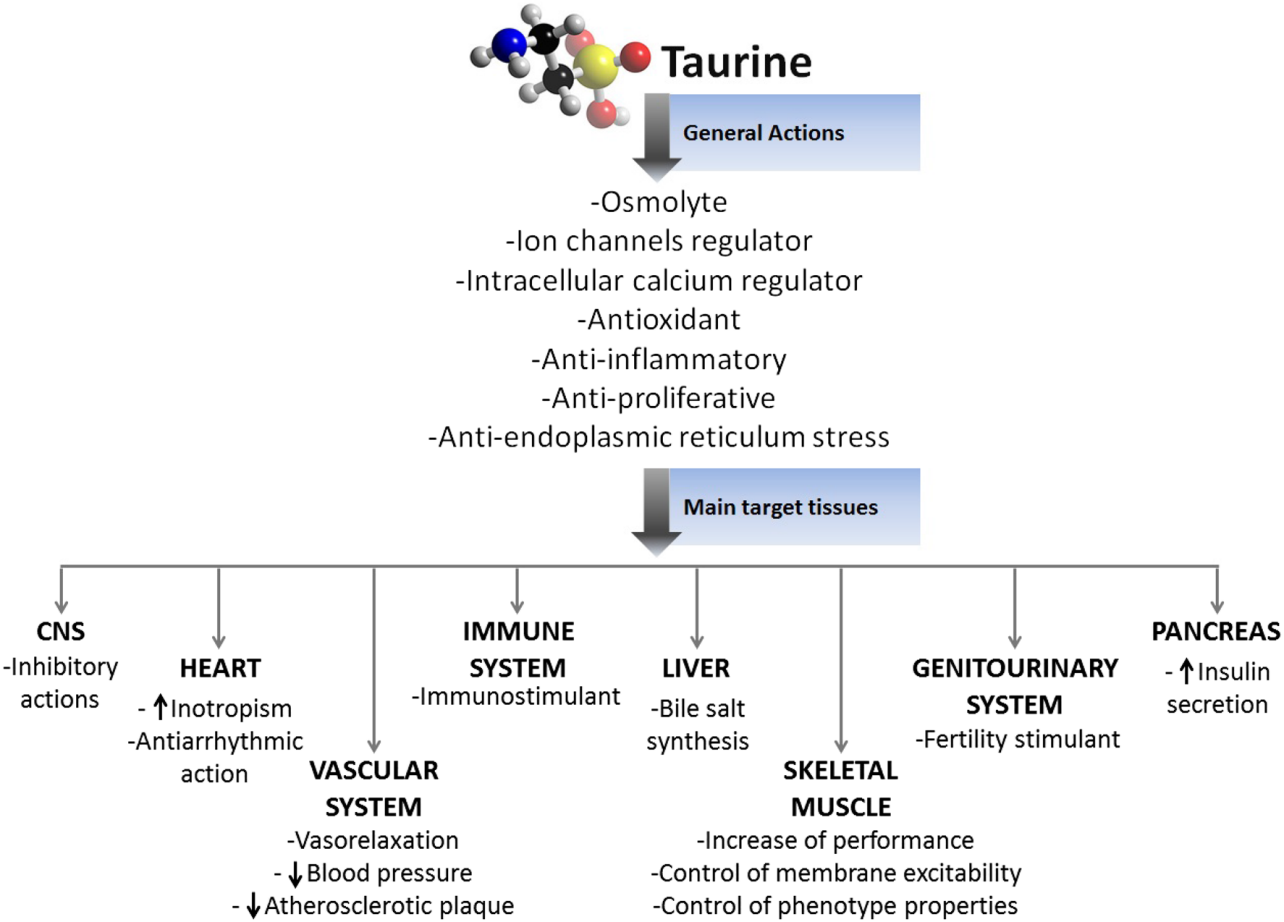
Taurine plays many and different physiological roles in various tissues. Some taurine actions, as the inhibitory effect at CNS, seem to be mediated by a receptor mechanism, while the effects on other tissues and systems occur via less defined mechanisms of action. Accordingly, the figure also briefly summarizes the main taurine effects ranging from control of calcium handling mechanism and excitation–contraction coupling in the heart, the ability to control immune reaction and inflammation, via inhibition of NF-kB as well as the main role of taurine in conjugating bile salts. Virtually all tissues are sensitive to taurine action with described effect of taurine on visual function (not shown), fertility, insulin release etc. The reported scheme is not supposed to be exhaustive of all taurine effects and only serves as general overview.

An extensive revision of all the actions of taurine in various tissues and the wide potential usefulness of its supplementation is out of the scope of this review. However, a general overview is provided in Fig. 2. As far as inherited or acquired pathophysiological conditions of skeletal muscle are concerned, the pre-clinical findings allow to distinguish effects related to exogenous pharmacological action of taurine on rather specific targets, such as in myotonic syndromes, to conditions that may be accompanied by changes in intercellular taurine content or change in calcium homeostasis, in which a taurine supplementation may be helpful to restore altered levels.

The present review is aimed at providing the state-of-art of taurine research in skeletal muscle, with particular attention to its potential therapeutic application as orphan drug in inherited rare muscle disorders, as well as in pathophysiological conditions such as aging, malnutrition and/or muscle disuse.

## Skeletal muscle ion channels as specific targets of taurine: the potential action of taurine as anti-myotonic drug

### Taurine and skeletal muscle chloride channels ClC-1

In CNS, taurine has been long claimed to act as an “inhibitory” amino acid and neurotransmitter [1]. Neuronal synthesis of taurine and metabotropic taurine receptors have been described in specific areas of CNS, where taurine acts in a glycine or GABA-like manner, by enhancing hyperpolarizing chloride-mediated conductance in nervous cells [9, 11, 12]. Pre-clinical evidences were provided of a beneficial effect of taurine in controlling/preventing seizure discharges and neurotoxicity [1, 12, 24]. The ability of taurine to act as inhibitory amino acid raised attention to its possible effect as potential membrane stabilizer in skeletal muscle. We investigated about the actions of the amino acid on voltage-gated chloride channels CLC-1 that account for the macroscopic chloride conductance (gCl) of skeletal muscle. Resting gCl accounts for about 70–90% to the total membrane conductance of sarcolemma and plays a pivotal role in maintaining the sarcolemmal electrical stability by shunting the depolarization-driven potassium accumulation in transverse tubules. Thus the large gCl allows repolarization and muscle relaxation.

Loss-of-function mutations of CLC-1 are responsible of myotonic syndromes with either autosomal dominant (Thomsen disease) or recessive pattern of inheritance (Becker’s Myotonia Congenita). The resulting decrease of gCl is responsible for the pathological hyperexcitability and for the delayed relaxation, spasms and stiffness typical of the disease in both patients and myotonic animals [25–27].

Our research has shown that taurine, acutely applied in vitro, exerts a concentration-dependent increase of gCl in rat extensor digitorum longus (EDL) myofibers, and in parallel reduces membrane excitability [28, 29]. The effective concentrations are in the millimolar range, likely in relation to the high intracellular level of the amino acid [28, 29]. A pre-clinical evaluation of the potential anti-myotonic activity of taurine has been performed. We found that taurine does not antagonize the myotonic discharges in rats made myotonic by administration of anthracene-9-carboxylic acid, a direct chloride channel blocker, nor does it restore gCl lowered in vitro by the same agent. However, when rats are made myotonic by a chronic exposure to 20,25 diazacholesterol, which reduces gCl indirectly by modifying lipid membrane composition, taurine antagonizes the electromyographic signs of myotonia if administered in vivo, while its acute in vitro application contrasts both the reduced gCl and the high frequency firing of single myofibers [30]. These results suggested that taurine can contrast myotonia if chloride channels are available for a direct modulation, implying its direct action at channel level or on a site nearby. A series of taurine analogues were tested on gCl of rat EDL myofibers to investigate the structure–activity relationship (SAR) between taurine and chloride channels. The results provided a pharmacological evidence of the presence of a specific low-affinity taurine binding site able to modulate chloride channel function and/or kinetic [31]. In particular, an increased distance between the two charged heads of taurine and/or a more distributed positive charge for the replacement of the amino group with aza-cyclo moieties lead to a decreased potency in enhancing gCl [31]. The direct action of taurine on skeletal muscle chloride channel was further confirmed by two microelectrode voltage-clamp recordings of chloride currents sustained by human ClC-1 channel heterologously expressed in Xenopous oocytes. In these conditions, the in vitro application of 20 mM taurine enhanced by 100% the chloride currents and shifted channel activation toward more negative potentials, an effect that likely accounts for the increase in resting gCl observed in native fibers [32–34]. This direct modulation adds to other possible homeostatic and modulatory roles that the high intracellular taurine has on chloride channels. However, as anticipated, the acute modulation of gCl may require fully or partly functional chloride channels, questioning about the real efficacy of taurine in ClC-1 related myotonic syndromes, especially for those mutations that seriously affect channel expression and protein level. Taurine has been tested in patients with myotonic dystrophy with encouraging results. In particular acute parenteral administrations of taurine allowed to reduce membrane excitability evaluated in relation to potassium plasma concentration after potassium-enriched infusion, suggesting again an action on membrane ionic conductance. Accordingly, a double-blind oral administration of taurine led to a long-term control of myotonic symptoms estimated as reduction of electromyographic (EMG) discharges and potassium induced-hyperexcitability [35–37]. Even taking into account the possible bias deriving from these small sized trials, the effects of taurine in myotonic dystrophy patients suggest alternative modality for decreasing membrane excitability. In fact, myotonic dystrophy type 1 (DM1) or Steinardt syndrome, is caused by expansion of a CTG trinucleotide repeat in the non-coding region of DM protein kinase with abnormalities in mRNA metabolism and alternative splicing of certain genes. In DM1 patients, the abnormal inclusion of alternative exons 6B and/or 7A and retention of intron 2 of CLC-1 channel gene (*CLCN1)* gene have been observed. These aberrant-splicing, which may also occur in myotonic dystrophy type 2 (DM2) patients, leads to premature termination codons, with a consistent decrease of the mRNA of *CLCN1*, of ClC-1 protein and consequently of gCl [38, 39]. Therefore, the possible modulatory action of taurine on other skeletal muscle ion channels has to be taken into account.

### Taurine and Nav1.4 voltage gated sodium channels

It is feasible to hypothesize a modulation by taurine of the skeletal muscle isoform of voltage-gated sodium channel (Nav1.4), involved in the generation and propagation of action potential and main target of symptomatic anti-myotonic drugs [37, 40]. The effect of taurine on sodium channels of native muscle fibers has been investigated in our laboratories by cell-attached patch clamp recordings. Taurine has a dual effect. In particular taurine enhances the sodium transients elicited by depolarizing test pulses close to the threshold for channel activation (test pulse to −70/−50 mV), an effect that is likely related to the observed shift of the activation curve towards more negative potentials. However, taurine reduces sodium currents at more depolarized test pulse potentials, with a 50% inhibition of the maximal peak sodium current observed at 10 mM taurine. In parallel, a left-shift of the steady-state inactivation curve has been observed, indicating the ability of taurine to stabilize the blocked channels in the inactivated state [34, 41 Desaphy and Conte Camerino, unpublished observation]. This peculiar effect of taurine on Nav1.4 channel is similar to what has been observed on cardiac sodium currents [42, 43] and underlines a complex action of the amino acid on sodium channel gating and kinetic. Our extensive structure–activity relationship studies of inhibitors of Nav1.4 channel allow to predict that the anesthetic-like action of taurine is mediated by the amino group, a main pharmacophore moiety in sodium channel blockers [44–47]. The dual ability of taurine to open chloride channels and to block sodium channels envisages a greater therapeutic action of the amino acid in myotonic states related to gain-of-function mutations of sodium channels, such as Sodium Channel Myotonia and Paramyotonia Congenita. The verification that taurine is able to compensate mutation-related biophysical alterations of Nav1.4 channels will be helpful at this regard, and is part of future projects of our laboratory. For the moment, the action of taurine on sodium channels can account for the antimyotonic effect in conditions where chloride channels are defective or dysfunctional [35, 36]. In line with this, the mechanism of taurine action on Nav1.4 sodium channels deserves to be further investigated since it may better support its pharmacological potential and its clinical use in hyperexcitability muscle disorders (Table 1).

**Table 1:** Involvement and therapeutic potential of taurine in physio-pathological conditions and diseases of skeletal muscle

Condition	Change in Taurine content /TauT	Pathogenetic mechanisms related to changes in taurine content	General symptoms	Taurine targets	Therapeutic Potential of Taurine
Post-natal development	Age-dependent increase in TauT expression and intracellular content	Delayed development and delayed acquisition of specific phenotypic properties; metabolic dysfunction	Specie-specific (due to different sensitivity to taurine deficiency)	Mitochondria; ion channels; calcium homeostasis and calcium dependent gene expression	Taurine supplementation in formula for pre-term born infants; to ensure a proper skeletal muscle phenotype differentiation
Aging	Decrease in Taurine content; no information on TauT expression	Metabolic distress; calcium dependent dysfunction; reduced regenerating ability; reduced activity of free-oxygen radicals scavengers	Sarcopenia; atrophy, weakness and fatigue degeneration, altered excitation–contraction coupling, impaired performance	Ion channels; Calcium homeostasis; oxidative stress and atrophy	To counteract the decrease in taurine content and the consequent reduction in chloride channel function and the alteration in calcium ion homeostasis; to ameliorate performance and muscle strength
Ischemia and reperfusion injury	Decrease due to a compensatory taurine efflux	Insufficient vaso-dilation in relation to muscle work; metabolic distress; oxidative stress	Hyperkaliemia, muscle dysfunction; ROS-induced inflammation and damage	Metabolic-sensitive channels; mitochondria	To counteract hyper-kaliemia by inhibiting K_ATP_ and KCa^2+^ channels; to prevent ischemia-induced taurine loss
Myotonic syndromes and periodic paralyses	Unknown	Primary inherited channelopathies due to loss-of function mutations of ClC-1 chloride channel or gain-of-function mutations of Nav1.4 sodium channel	Hyperexcitability and impaired muscle relaxation	ClC-1 chloride channel; Nav1.4 sodium channel	To reduce membrane hyper-excitability through: opening of chloride channel and increase in gCl mediated by both short and long term actions; modulation of generation and propagation of action potential, by blocking sodium channel with a local-anesthetic like mechanism
Disuse	Slow-to-fast decrease in taurine content; no change in TauT expression	Myofiber phenotype transition in postural muscle; atrophy	Atrophy, change in metabolism, slow-to-fast transition; weakness	Ion channel function and expression; calcium homeostasis	To counteract disuse-induced taurine loss; to counteract myofiber transition; potential counteraction of atrophy
Duchenne muscular dystrophy and related myopathies	Change in content related to pathology phase; possible reduction of TauT expression	Alteration of calcium homeostasis; calcium-related degeneration; oxidative stress and inflammation	Progressive muscle degeneration and weakness; muscle fiber loss and fibrosis; sarcolemmal instability; altered calcium homeostasis; inflammation and oxidative stress	Chloride channel and voltage-insensitive calcium permeable channels (Leak/TRP-like); SERCA; mitochondria	To ameliorate muscle performance; to counteract taurine loss and to modulate calcium availability for contraction; to counteract contraction-induced ischemia. To contrast degeneration-induced decrease in gCl; adjuvant therapy in combination with glucocorticoids

The table summarizes the main role of taurine in various conditions of skeletal muscle, indicating evidences in relation to changes in tissue content and potential site of taurine action. Please refer to text for more detailed information and specific references.

*TauT* taurine transport system, *SERCA* sarco/endoplasmatic reticulum calcium ATPasi, *gCl* macroscopic chloride conductance, *TRP* transient receptor potential channels, *ROS* reactive oxygen species, *KATP* ATP-dependent potassium channels, *KCa* calcium activated potassium channels.

## Role of proper taurine intramuscular level for excitation–contraction coupling and muscle performance

The ability of skeletal muscle to concentrate taurine against gradient pushed toward a better understanding of its physiological role. Adult rats were chronically treated with guanidinoethane sulfonate (GES), an inhibitor of taurine transporter (TauT) to induce a reduction of taurine content in skeletal muscle. We found that a 50% reduction of taurine in EDL muscle leads to a marked decrease in gCl, and to a parallel enhancement of sarcolemmal excitability, disclosing the ability of taurine level to exert a physiological control on chloride channel function and sarcolemmal stability [48]. The mechanism underling this effect is not clear yet, but we cannot rule out the ability of taurine to modulate ClC-1 channel function via a fine-tuning of a calcium-dependent phosphorylation-signaling pathway, as discussed below. In line with the described ability of taurine to control calcium homeostasis in both skeletal muscle and cardiac tissue [1, 4], we found a marked alteration of mechanical threshold, i.e. the voltage at which muscle fiber contracts in response to depolarizing voltage steps, in taurine-depleted EDL myofibers. Mechanical threshold depends on the kinetic of calcium release from and reuptake by sarcoplasmic reticulum, also in relation to basal cytosolic calcium concentrations. Taurine depleted EDL muscle fibers contract at more negative potentials with respect to normal ones, implying an impact of GES treatment on calcium handling [48, 49]. Both the decrease in gCl and the shift of mechanical threshold toward negative potentials were rapidly reverted by in vitro application of millimolar concentration of taurine. Actually, depleted muscles showed a higher than normal sensitivity to exogenous taurine with respect to normal ones [48], further corroborating the link between the observed alterations and the taurine level. The contractile properties and fatigability of EDL muscles depleted of taurine by a GES treatment were investigated by Bakker’s group. It was found that the treatment with GES decreases muscle taurine levels to <40% of controls and decreases the peak twitch force of EDL muscles by 20%. Also, GES-treated muscles develop a lower force in force–frequency relationship and show a slower time to fatigue, likely in relation to the lower metabolic demands of the weaker muscles [50]. Primary information about the long-term effect of taurine in skeletal muscle and, consequently, of potential usefulness of its exogenous administration derives from studies on mice in which the TauT was genetically knocked out [6, 14–16]. TauT knockout mice (TauT^−/−^) show more than 90% decrease in taurine content in both muscle and heart and are characterized by a marked decrease in exercise performance in exhaustive training models. Although the force of isolated muscle has not been measured in these TauT^−/−^ mice, clear abnormalities of muscle structure have been found, including signs of atrophy and muscle necrosis. Additionally, the muscles of TauT^−/−^ mice have a shift of metabolism toward the glycolytic pathway, especially in condition of exercise; this has been related to a dysfunction in mitochondrial function and in fatty acid oxidative pathways [51]. In parallel, taurine deficiency leads to cardiomyopathy characterized by remodeling of ventricular cardiomyocytes, ultrastructural damages of myofilament and mitochondria, and overexpression of markers of heart failure, such as atrial natriuretic peptide, brain natriuretic peptide and beta-myosin heavy chain [15, 16].

It is therefore evident that taurine is essential to maintain muscle performance and excitation–contraction coupling; however the mechanism for these actions is still unclear. An in vitro study of Berg and Bakker clearly demonstrated the ability of taurine to increase the accumulation of calcium into sarcoplasmic reticulum (SR) in isolated skinned myofibers by 35%, an effect that accounts for the greater depolarization-induced contraction of fiber exposed to 20 mM taurine. This in spite taurine slightly reduces the sensitivity of contractile apparatus to calcium [52]. Interestingly, a recent study demonstrated that a prolonged exposure to 10–20 mM taurine increases the rate of calcium uptake in both type I and type II human myofibers; an action within the SR lumen has been proposed. An increase in contractile sensitivity to calcium was also observed but exclusively in type I fibers [53]. These results reinforce the original data of Huxtlable and Bressler about the ability of taurine to stimulate calcium uptake by vesicles of SR [13]. Recent insight into the role of taurine in skeletal muscle has been obtained by the group of Hayes, who supplemented rats with taurine and evaluated the outcome on various functional parameters [54]. Taurine supplementation significantly increases the amino acid content in skeletal muscle, without any adaptive change in TauT activity; in parallel an increase in force and a greater resistance and recovery after fatigue have been observed. These changes were paralleled by an increase in calsequestrin1, the calcium binding protein that works to maintain high amounts of calcium in the cysterna of SR. This suggests that taurine supplemented muscle can store a greater quantity of calcium with a consequent greater calcium availability for contraction. However, the involvement of sarco/endoplasmic reticulum calcium-ATPase (SERCA) remains to be better clarified. A decrease in markers of oxidative stress was also found, indicating that taurine may help to control activity-related oxidative stress [48]. In support to this view, a recent report by Silva et al. showed that a daily treatment of rats with 300 mg/kg taurine for 2 weeks protects muscles against in vivo eccentric exercise damage, such as downhill running [55]. In particular taurine reduced protein carbonylation or oxidized thiols, without increasing the expression of endogenous anti-oxidant pathways, such as superoxide dismutase or catalase [55]. Sugiura et al. similarly found that taurine administration before strenuous exercise reduces muscle DNA damage likely via down-regulation of inducible nitric oxide synthase (iNOS) and consequent reduction of nitrosative inflammation [56]. The protective effects of taurine supplementation are due to a long term modulatory effect, likely in relation to its muscle uptake and intracellular levels. In fact acute in vitro application of physiological concentrations of taurine to isolated mouse soleus muscle, does not increase muscle contractile performance in term of force, fatigue resistance and recovery and does not exert any synergistic action when associated with caffeine [57]. Despite the authors suggesting a lack of ergogenic benefit by acute taurine, it is important to underline that slow twitch soleus muscle is characterized by high intracellular taurine content [58, 59], predicting its lower dependency on extracellular concentrations. Accordingly, we have shown that a chronic treatment with taurine to dystrophic mice leads to a minor increase of its intracellular content in soleus muscle than in fast twitch muscles [59].

Although taurine supplementation enhances exercise performance, its efflux during exercise and/or ischemia, with consequent decrease in tissue concentration, can also occur [60, 61]. Whether the loss of taurine is a marker of tissue damage or rather a cytoprotective mechanism against ischemic insult, is still matter of debate [60, 62, 63]. The protective effect of taurine efflux in the above conditions can be related to the need to osmotically balance, along with water movement, the increase of by-products of metabolism in the myofibers [1, 14]. However a role in the mechanism to contrast fatigue can be envisaged. In fact, taurine exerts an inhibitory control on channels that couple the metabolic state of the myofiber with membrane excitability, such as the ATP-dependent potassium (KATP) channels and calcium-activated potassium channels [64, 65]. Taurine blocks skeletal muscle KATP channel by binding the channel complex nearby the sulphonylurea receptor [64]. During ischemia–reperfusion injury, the opening of KATP are involved in the cytoprotective effect of the preconditioning mechanisms, by preventing the influx of calcium ions and preserving the ATP content of the muscle. The efflux of taurine during exercise and/or ischemia may be required to relief a basal inhibitory effect and to enhance the potassium efflux and membrane repolarization via the specific channels activated by ATP depletion and/or intracellular calcium accumulation. This would exert a protective action against exercise-induced fatigue or impairment in muscle performance related to ischemia–reperfusion injury [64, 65]. Accordingly, the depletion of taurine induced by GES in rat skeletal muscle significantly increases the macroscopic resting potassium conductance of about 80% [48].

**Fig. 3:**
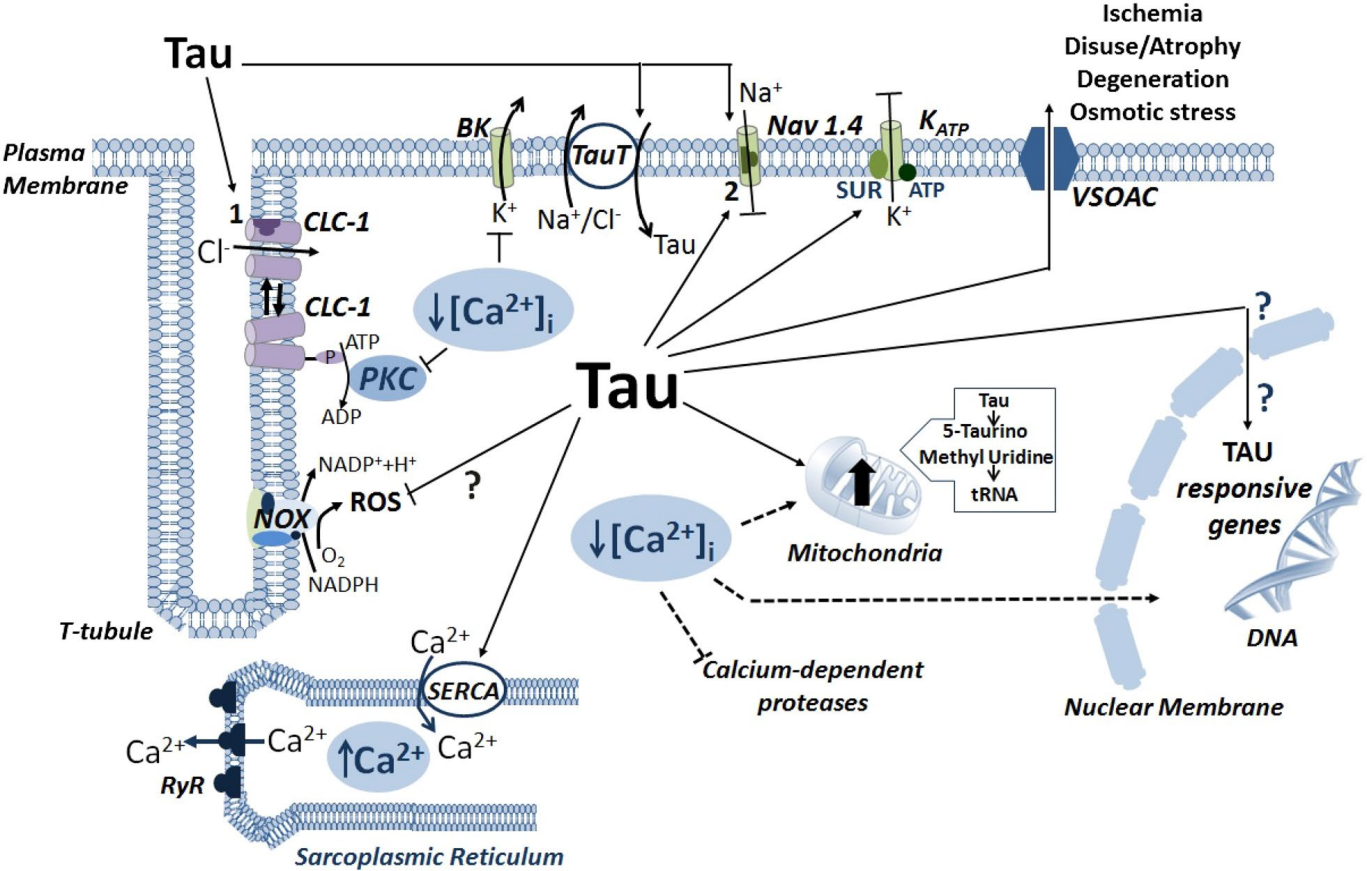
Representative scheme of the action of taurine in skeletal muscle fiber. It is shown the TauT carrier which works to maintain high intracellular taurine level, along with the actions that taurine exerts on membrane channels, sarcoplasmic reticulum, mitocondria and possibly gene expression. Putative binding sites for taurine are shown (1) on ClC-1 channel and (2) as local anesthetic drug binding site. *Arrows* indicate a general stimulating action while *dotted lines* are for inhibitory effects or yet undefined pathways. A pathway for taurine efflux under stress conditions (ischemia, osmotic stress, etc.) likely via the volume-sensitive organic anion channel (VSOAC) is also shown.

Intracellular taurine can also be conjugated in mitochondria of extra-hepatic tissues to 5-taurinomethyl uridine that is present in tRNA and modulates the synthesis of mitochondrial proteins. Consequently, the fatigue and the enhanced oxidative stress observed in myopathic states by taurine depletion can also be due to respiratory chain inefficiency [4, 51, 66]. A representative scheme of the taurine actions in striated myofibers is shown in Fig. 3.

## Taurine as potential therapeutic muscular agent from birth to elderly

The role of taurine for post-natal development of various organs depends upon the species-specific ability to endogenously synthesize the amino acid. Cats, that critically depend on exogenous taurine intake, develop serious impairments during post-natal development if not fed with taurine. Although less compelling for humans, prematurely born infants are believed to lack the enzymes that convert cystathionine to cysteine, and may, therefore, become taurine-deficient if not breast-fed. In fact taurine is present in mother’s milk and evidences are available about potential usefulness of taurine addition in the formula especially for pre-term births [67, 68]. The actual necessity or benefit of this practice has never been rigorously studied, and as such, taurine has yet to be proven to be important during fetal development, perhaps via epigenetic and/or organogenesis related mechanisms. Recent focus has been addressed to the potential benefit of taurine supplementation in mice during gestational period, especially when mothers are exposed to low-protein diet, a condition mimicking the low weight at birth and related to the risk of developing dysmetabolic states later on [69]. In these conditions taurine protects pancreas by decreasing islet sensitivity to cytokines and shows to have an impact on gene expression and “reprogramming” in various tissues, including skeletal muscle [70–72].

In support of the pivotal role of adequate taurine level for skeletal muscle development, we demonstrated that taurine muscle level increases during the first month of rat post-natal life [73]. This increase matches the acquisition of phenotype-specific contractile properties. In particular in rat fast-twitch EDL muscle it occurs in parallel with the post-natal increase in muscle gCl and of ClC-1 channels expression; i.e. during the acquisition of the mature profile [39, 73–75]. Adult levels are likely to be attained later, since a proton nuclear magnetic resonance (H-NMR) study showed an increase in taurine in different rat skeletal muscles from 6 to 18 weeks of age [76]. Accordingly, an age dependent increase of taurine as well as of other amino acids, has been found in muscle of metabolically healthy children (age range 1–15) with respect to adults [77].

In agreement with an active role of taurine for muscle phenotype acquisition, supplementation of mothers during pregnancy and lactation as well as of new-born rats results in a higher content of the amino acid in skeletal muscle, accompanied by a more rapid development of gCl [73]. Whether such an increase is due to a modulatory action of taurine on ClC-1 channel or to an effect on its gene expression is not known yet. Importantly, a profound alteration in gene expression has been described in liver and skeletal muscle of pups that were exposed pre-natally to low protein diet, while the addition of taurine to mothers via drinking water during gestation leads to a marked protection [71, 72]. Focusing on skeletal muscle, the rescuing effect of taurine did occur for genes involved in oxidative phosphorylation and in the tricarboxylic acid cycle that were markedly down-regulated in skeletal muscle by the low protein diet. Importantly, plasma taurine concentration has been suggested to be a marker of fetal well-being and a prerequisite for normal fetal development [78]. In line with the important role of taurine for skeletal muscle development, the TauT expression increases during myogenesis and its gene has consensus site for myocyte enhancing factor 2 (MEF2), being therefore under strict control of myogenic program [79]. Also, taurine has been shown to stimulate myofiber differentiation in vitro [80]. Although the mechanism through which taurine may control gene expression during development is not clear yet, it appears to be a necessary factor in myogenesis, and perhaps in mitochondrial biogenesis, with key role for tissue development (Table 1).

Another condition that may benefit from taurine supplementation is aging. Age-related sarcopenia is accompanied by profound changes in hormonal and metabolic profile of skeletal muscle. An important alteration in the content of various amino acids occurs in human muscle specimen with age, as a result of age-related increase in proteolysis; in parallel a marked decrease in taurine content has been observed [81].

Besides sarcopenia, skeletal muscle of aged rats develops features that are overlapping those observed in taurine depleted muscles, i.e. a marked decrease in gCl and a change in calcium homeostasis with a shift of mechanical threshold towards more negative potentials [82, 83]. We found by high-performance liquid chromatography (HPLC) determination that muscle taurine concentration is in fact significantly decreased in muscle of aged rats; however the levels can be restored to adult values upon the exogenous administration of taurine for 3 months (1 g/kg in drinking water) [84]. Importantly, the taurine administration counteracts the decrease in gCl and the alteration in excitation–contraction coupling of aged rat EDL muscle, supporting the key role of the amino acid in the alterations observed and the potential beneficial role of its supplementation in elderly subjects (Table 1). In the EDL muscle of aged rats supplemented with taurine an almost complete recovery of the pharmacological sensitivity of gCl to either direct and indirect channel modulators, such as the enantiomers of p-chloro-phenoxy propionic acid and the phorbol esters, respectively, was observed. The effect of these latter, along with the amelioration of mechanical threshold observed, discloses the ability of taurine to modulate gCl by reducing the phosphorylation state of the chloride channel brought about by calcium and phospholipid-dependent protein kinase C [83, 84]. This offers a unifying mechanism for physiological taurine action via calcium homeostasis and modulation of calcium-dependent signaling pathways.

In line with the above observations, TauT^−/−^ mice show accelerated senescence, with greater muscular damage and endoplasmic reticulum stress due to accumulation of misfolded proteins. A central role of calcium mishandling has been proposed, along with the interest in maintaining adequate taurine level for contrasting aging-related muscle impairments [85].

## Taurine and muscular dystrophy

The alteration of calcium homeostasis is a hallmark of muscles affected by inherited muscular dystrophy, such as in mice with X chromosome-linked muscular dystrophy (mdx), the most widely used model for Duchenne muscular dystrophy (DMD). It is believed that the absence of dystrophin, a protein with a key role for sarcolemmal integrity and mechano-transduction, leads to sarcolemmal tears and to overactivity of voltage-insensitive cationic channels which enhance passive calcium entry, especially during work load [86–88]. This in turn leads to both the alteration of excitation–contraction coupling and to the activation of degenerative pathways [88, 89]. We have found that the EDL muscles of dystrophic mdx animals undergoing chronic exercise protocols, have features resembling taurine depleted ones, i.e. a reduction of gCl and a negative rheobase voltage for mechanical activation [89, 90]. Dystrophic muscle may have a reduced ability in retaining intracellular taurine; in fact we observed a trend of a lower than normal taurine muscle concentration in parallel with markedly high levels in plasma [89]. Accordingly, other authors found that taurine levels fluctuate in mdx muscles in relation to the disease phase, with compensatory increases being observed after acute degenerative period and glucocorticoid treatment [91, 92]. In this frame, taurine seems to be a useful marker of the dystrophic state of mdx mice when monitored by H1-magnetic resonance spectroscopy both in vivo and ex vivo, although technical problems may still limit the accurate peak resolution for quantitative evaluation [91–95]. In our experiments, the in vitro application of millimolar taurine concentrations fully restored the alteration of mechanical threshold observed in these animals [89]. Interestingly, similar results have been obtained upon chronic taurine treatment in exercised mdx mice. The in vivo treatment also significantly contrasted the decrease in gCl and lead to a significant increase of mouse strength in vivo, due to an interesting anabolic action of the amino acid in the dystrophic animals [90]. As previously mentioned, TauT^−/−^ mice are characterized by a marked 80% decrease in exercise performance and increased fatigability, a feature that is classically observed in the mdx phenotype [6, 14, 90, 96]. The role of taurine in muscular dystrophy is also under study in Hayes’ laboratory, where a lower expression of TauT in mdx mouse muscle has been demonstrated, which is not influenced by exogenous taurine administration [97], supporting the difficulty of dystrophic muscle to retain taurine. Exercise protocols may differently modulate intramuscular taurine concentration, ranging from no change to phenotype-dependent decrease, likely in relation to the exercise type; however taurine supplementation can enhance exercise performance [60, 61]. Due to the impaired mechano-transduction of dystrophic myofibers, it would be of interest to evaluate whether the exercise protocol in mdx mice can lead to a further distress in taurine concentration and in TauT expression; this is currently ongoing in our laboratory.

Based on first encouraging results, we tested the possible advantage to combine taurine with α-methylprednisolone, a glucocorticoids currently in use in dystrophic patients [58]. A synergistic action of the two drugs in enhancing mouse strength and in restoring calcium homeostasis was observed, with a normalization of mechanical threshold and a reduction of the over-activity of the cation channels likely involved in abnormal calcium entry [58, 86, 98]. The treatment was also associated with a significant increase in taurine content in fast-twitch limb muscles, suggesting that dystrophic muscle maintains the ability to uptake taurine if adequately supplemented [58]. The synergistic action observed corroborates a potential interest of taurine as adjuvant therapy in steroid-treated patients. This is also supported by the evidence that glucocorticoids exert an inhibitory action of renal taurine re-uptake, then leading to hypotaurinemia, which in turn may have long-term negative effects on cardiovascular function [5].

Importantly, the taurine treatment to mdx mice significantly reduces the high plasma level of lactate dehydrogenase, an index of metabolic distress, and it is worth to underline that a marked increase in plasma lactate actually occurs in TauT^−/−^ mice [6]. Therefore taurine can also play a role in metabolism in dystrophic muscle, similarly to what observed in exercise-challenged TauT^−/−^ mice [51].

Increasing evidences suggest a link between calcium homeostasis, oxidative stress and mitochondrial distress in muscular dystrophy, leading to reconcile all these taurine actions under few main mechanisms, although not fully clear yet [99, 100]. As already mentioned, taurine supplementation contrasts the exercise-induced increase in oxidative markers, without enhancing the level of endogenous anti-oxidant [55]. Other evidences support that the sulfonic amino acid is actually incapable of scavenging the common oxidants, namely, superoxide, hydrogen peroxide and hydroxyl radical, which instead are the main products of enhanced NADPH oxidase activity in dystrophic muscle [99–101]. However, the amino group of taurine can neutralize hypochlorous acid, one of the reactive species generated by myeloperoxidase-halide system in neutrophils [102]. In that reaction, taurine is converted to taurine chloramine, which is less toxic than hypochlorous acid and actually serves as a modulator of the immune system also by interfering with the production of several pro-inflammatory mediators and activation of the transcription factor nuclear factor kappa-light-chain-enhancer of activated B cells (NF-kB) [102]. In addition, taurine has been proposed to directly activate peroxisome proliferator-activated receptor γ (PPARγ) in epithelial cells, a mechanism that may account for its protective action against inflammation-related diabetic retinopathy progression [103]. In consideration of the involvement of chronic inflammation and NF-kB derived mediators in dystrophic muscle [87, 104, 105], the above immunomodulatory actions of taurine are of value. However, whether the anti-inflammatory and anti-oxidant action contributes to the beneficial effect observed in dystrophic animals is not known yet and the evaluation of biomarkers in samples of taurine treated mdx mice will be useful at this regard. Our preliminary results favor a decrease in superoxide anion formation, measured by dihydroethidium staining, in tibialis anterior muscles of exercised mdx mice treated with taurine (De Luca, personal unpublished observations). An attractive hypothesis, currently under study in our laboratory, is that taurine may contrast the impaired SERCA activity in dystrophic muscle either directly or by reducing the damaging effect brought about by oxidation and/or nitrosylation [13, 54, 106]. Interesting recent results of Terrill et al. have shown that a chronic administration of the cysteine precursor 2-oxothiazolidine-4 carboxylate (OTC) markedly decreases the level of thiol oxidation in muscles of mdx mice; in parallel an amelioration of force and muscle morphology has been observed. Importantly the administration was not paralleled by an increase in cysteine or glutathione but rather by an increase in taurine level. The authors underlined that the decrease in taurine content may have a direct causative role in enhanced susceptibility to oxidative stress, disclosing a novel mechanism for beneficial effect of the classical anti-oxidant *N*-acetylcysteine [107].

Considering the mitochondrial sufferance occurring in dystrophic muscle [93], the previously described role of taurine for preserving mitochondrial function has to be taken into account for further studies. Similarly, the potential role of taurine and its chemical chaperone conjugate tauroursodeoxycholic acid in contrasting endoplasmic reticulum stress in various conditions should be considered for the acute and chronic ability of taurine to modulate signaling pathways [108, 109]. In addition, taurine may improve muscle metabolism by contrasting functional ischemia, based on the described vasodilating properties [110]. The clarification of the mechanism of action and the evaluation of long term safety and efficacy also at heart level can add important pre-clinical data to plan clinical trials in DMD patients (Table 1).

## Taurine and disuse-related muscle atrophy

Muscle disuse is a general term which describes a condition of inactivity occurring after prolonged bed rest, spaceflight and/or aging. The slow-twitch muscles, devoted to postural maintenance, are the most affected ones, showing a slow-to-fast phenotype transition and severe atrophy, both leading to impaired muscle function. The adaptation of skeletal muscle to different activity includes changes in the expression of structural, metabolic and contractile proteins that fine-tune the characteristics of this tissue. The hindlimb unloaded (HU) model of disuse in rodents is a widely accepted ground-based model that mimics microgravity condition and is used to study the mechanisms responsible for the disuse-induced modification of skeletal muscle function. The soleus muscle of HU rats and mice becomes atrophic and experiences a slow-to-fast phenotype transition, characterized by an increased expression of the fast myosin heavy chain (MHC) isoform [111, 112]. Along the years, the studies on the HU model have shown that various proteins involved in the control of sarcolemma excitability, calcium ion homeostasis, energy metabolism, and contractile machinery undergo changes in the expression, turnover, and activity in accord with the entering of the slow muscle into a fast program [111, 113–117]. In particular, ClC-1 chloride and Nav1.4 sodium channels are differently expressed in fast-twitch and slow-twitch skeletal muscles, the expression of both being higher in the former. Accordingly with the change of phenotype, ClC-1 channel activity and expression as well as the intracellular resting calcium level in slow-twitch soleus muscle are significantly shifted by HU process toward the values of a fast muscle, even before the modification of MHC expression [111]. Similarly, HU increased sodium current density and sodium channel mRNA level in soleus muscle fibers [113]. All these changes alter the resistance to fatigue of antigravity muscle fibers, an effect that may contribute to the impairment of muscle function, in terms of excitability and contraction. A full understanding of the mechanisms of disuse-induced muscle alterations in humans is still incomplete and few molecules have been proposed for therapy [118, 119]. However, supplementation with essential amino acids and carbohydrates in combination with exercise attenuates muscle protein loss in humans exposed to prolonged inactivity [120, 121]. Based on these considerations and on our previous findings about the action of taurine in the modulation of calcium homeostasis and ion channel function [34, 41, 49], we focused on taurine as a potential candidate to counteract the HU-induced phenotype transition and skeletal muscle function impairment [1, 34].

In agreement with a critical role of taurine in phenotype-specific cellular function, the concentration of the amino acid is twofold higher in soleus compared to EDL muscle. The physiological relevance for this phenotypic difference is still unknown but various hypothesis can be raised based on the essential role of taurine in skeletal muscle and its actions in metabolism and phenotype-dependent properties. Interestingly, our recent findings [59] showed for the first time a marked reduction of taurine content in the soleus muscle of HU rat. This muscle loss would be consistent with an original report of National Aeronautics and Space Administration (NASA) describing a large excretion of taurine in the urine of the astronauts of the APOLLO mission [122]. In spite of the reduction of taurine in soleus muscle of HU rats, the expression of TauT was unchanged. Indeed, TauT expression was found to be higher in slow-twitch soleus muscle with respect to the fast EDL, and was not reduced during HU, suggesting that the intracellular reduction of taurine is not associated with the change of phenotype. In addition, our data suggest that TauT activity is efficiently maintained during HU, since taurine oral supplementation fully prevents the loss of taurine content in HU-soleus muscle. Thus, we hypothesize that the reduction of intracellular taurine content during HU is likely due to increased taurine efflux. A possible explanation might be that taurine leakage compensates for intracellular osmolarity changes, which likely occurs due to muscle protein degradation and increased catabolism. Accordingly, the production of intracellular osmolytes during muscle disuse atrophy has been described, which may justify taurine escape in this condition [123–125]. Importantly in rats fed with taurine, TauT expression was reduced in soleus muscle, suggesting a negative feed-back regulation as a mechanism to control taurine intracellular level. As anticipated the TauT expression is under control of MEF2, a determinant of slow-fiber phenotype [79], thus it is tempting to speculate that TauT expression after taurine supplementation can be reduced by a mechanism involving a complex cross-talk between taurine and ClC-1 modulation during the phenotype transition.

Our findings also highlighted that taurine supplementation in HU rats has preserved resting gCl and resting cytosolic calcium level together with the slow MHC phenotype in the soleus muscle.

However, taurine had little effect on muscle atrophy, which is a severe condition occurring during HU as well as in various muscle diseases [126]. Indeed, it did not prevent the reduction of muscle-to-body weight ratio and of the fiber cross sectional area (CSA), while it partially contrasted the expression of atrogin-1 and mostly of muscle RING-finger protein-1 (MURF-1), two ubiquitin–proteasome pathway enzymes, that are strongly up-regulated as a result of HU-induced atrophy [127]. Such an effect suggests that a longer treatment or a different therapeutic schedule of taurine might have protective effect against muscle atrophy and might be useful to reach a complete muscular recovery. However complex mechanisms control the relative expression of atrogin and MURF-1 in skeletal muscle under various insults [79, 128] and further experiments are needed (Table 1).

## Taurine and human skeletal muscle

Taurine has limited use in clinical settings although human use has been considered for specific diseases such as non-insulin dependent diabetes and related disorders, to treat alcohol withdrawal, congestive heart failure and arrhythmias, rheumatoid arthritis and other chronic inflammatory states, seizure disorders, and liver related disorders [19, 102, 129]. In Table 2 is a brief report of some clinical studies related to taurine supplementation, with relative dosages and outcomes. Most of them focused on diabetes mellitus, insulin resistance and diabetic complications, based on the rationale that plasma taurine concentration is reduced in patients with insulin-dependent diabetes mellitus (IDDM) [129–136]. Taurine was indicated in addition to specific drugs. Other clinical studies tested taurine in congestive heart failure, hypertension, inherited succinic semialdehyde dehydrogenase deficiency, obesity or its supplementation in aged individuals [137–143].

**Table 2:** Clinical use of taurine in different pathophysiological conditions

References	Patients	Dose (g/day or mg/kg)	Duration	Result
Franconi et al. [130]	IDDM (Diabetes mellitus type 1)	1.5 g	90 days	No effect
Elizarova and Nedosugova [131]	IDDM	1 g	30 days	Glucose metabolism and trygliceride level improved
Chauncey et al. [133]	NIDDM (DM type 2)	3 g	4 months	Plasma taurine level increased
Brøns et al. [134]	Overweight non-diabetic	1.5 g	8 weeks	No effect
Xiao et al. [136]	Overweight non-diabetic	3 g	2 weeks	Insulin sensitivity improved
Nakamura et al. [132]	NIDDM with microalbuminemia	3 g	12 months	No effect
Moloney et al. [135]	IDDM	1.5 g	2 weeks	Endotelium-dependent reaction improved
Gonzales-Contreras et al. [142]	Cholestasis by parenteral nutrition	~25 mg/kg/day	~50 days	Hepatoprotection with reduction of AST, ALT and GGT
Rosa et al. [143]	Obesity	3 g/day	8 weeks	Increase in plasma levels of taurine and adiponectin; reduction of inflammatory markers
Pearl et al. [141]	Succinic semialdehyde dehydrogenase deficiency (efficacy, safety and tolerability)	50–200 mg/kg/d (age range 12 years)	13 months (mean time from 3 to 50)	No significant effects Tolerability issues at highest doses
Fujita et al. [139]	Hypertension	6 g	7 days	Systolic and diastolic pressure improved
Azuma et al. [138]	Congestive heart failure	6 g	4 weeks	Heart parameters improved
Bergamini et al. [137]	Epilepsy	200 mg–21 g	Various	Seizure frequency reduction
Durelli et al. [36]	Dystrophic myotonia	6–10 g	6 months	Myotonic symptoms improvement
Dunn-Lewis et al. [140]	Elderly	500 mg in multinurtient supplement	4 weeks	Physical function improved

A part for the use in myotonic dystrophy patients [35–37], the potential therapeutic role of taurine for skeletal muscle disorders has yet to be verified in clinical settings. In fact, most of the studies about the role of taurine for skeletal muscle physiology and its potential in pathological conditions have been carried out in animal models. In these conditions taurine depletion or supplementation are directly correlated with changes in the amino acid content in skeletal muscle, which facilitate the drawing of conclusion about amino acid action and potential. However, few studies have been conducted in humans, and some contradictory reports are available, questioning about the actual usefulness of taurine supplementation or on its mechanism of action. Apart for the age-related changes reported in the previous paragraphs, one of the main issue concerns the modulation of taurine concentration in adult skeletal muscle under conditions of exercise and/or metabolic distress. Galloway et al. [144] demonstrated that taurine supplementation to exercised healthy adults leads to a marked increase in the amino acid plasma level that however is not paralleled, after 7 days of supplementation, by an increase in skeletal muscle. They proposed that intramuscular taurine concentration is tightly regulated and that high plasma level may actually work to reduce TauT activity in order to maintain constant the amino acid level. Therefore, even chronic oral taurine supplementation may cause less increase in human muscles than in rodent ones, and the observed muscle effects could be due to extracellular taurine actions. In addition, plasma levels are also tightly regulated via overexpression of TauT in kidney, which may also show specie-specific regulatory pathways [145, 146].

The dose is another important issue. In fact murine pre-clinical studies often require about tenfold higher concentration that in human trials; by the way this has to match the endogenous high level of taurine in target organs. In addition, an accurate muscle exposure to taurine after oral ingestion requires a careful assessment of the pharmacokinetic profile that has not been extensively evaluated in humans. In line with Galloway et al. [144], a single oral dose of 4 g in healthy volunteers allows to get a maximal plasma peak in about 1.5 h and showed an half-life of 1 h with a first-order kinetic clearance; this is in line with kidney being the main organ regulating taurine level [147]. Generally the daily dose of taurine ranges between 3 and 6 g; consequently its fast kinetic can account for some of the puzzling data obtained, suggesting the need of a more careful determination of the optimum dose. It is important to underline that most of the available evidences focus on the usefulness of taurine supplementation in sustaining muscle function in trained individuals. Balshaw et al. have recently evaluated the outcome of 1 g taurine ingestion, evaluated in blind against placebo, on running performance of trained middle-distance runners. They described a modest, although significant, increase in performance in the taurine-treated group, without any change in metabolism parameters [148]. The authors claimed that a similar improvement of performance after taurine ingestion, without changes in oxygen uptake or plasma lactate, has been found in other studies [144]. Taurine muscle levels were not assessed, thus the correlation between taurine effect and a specific muscle action is rather indirect. Accordingly, they speculated about alternative potential mechanisms, such as the action of taurine at muscle membrane level, in preventing taurine drop during exercise or rather an effect on neuronal function.

In another study, a combination of taurine (2 g) and branched-chain amino acids three times a days for 2 weeks before eccentric exercise, plus 4 days after, has been tested in healthy untreated volunteers. The eccentric exercise protocol consisted of repeated sets elbow flexion at 90° to an extended position, finally leading to uncontrolled damaging stretch. The combination exerted a greater protection against muscle damage and delayed-onset muscle soreness than single administrations, although no detailed investigation has been done to clarify the mechanism of action and/or the amino acid level into the muscle [149]. Similarly, da Silva et al. have recently described the ability of 14 days taurine administration to increase strength of the elbow flexor subjected to eccentric exercises in young adult males; in parallel, markers of oxidative stress were reduced, without increase in endogenous anti-oxidant expression nor changes in inflammatory markers. Again muscle taurine level were not determined [150]. Therefore the available evidences do not allow to conclude about the ability of supplemented taurine to actually increase its muscle level in adult healthy and trained individuals, suggesting alternative modality of action, i.e. at neuromuscular system. However, it cannot be ruled out that taurine supplementation may effectively enhances muscle taurine levels in conditions characterized by more dramatic fluctuation of its content. This applies to postnatal development and aging, and mostly to pathological conditions such as muscular dystrophy and disuse-related muscle dysfunction (Table 1) [151]. More direct evidences in humans and patients will be helpful, in order to better correlate the effect of exogenous administration of taurine with the ability of residual muscle tissue to uptake the right amount, or rather to disclosure taurine actions independent on its intracellular levels [145]. In addition, an inter-individual variation in plasma increase of taurine after supplementation may occur in relation to both nutritional state, age, drug interaction, while gene polymorphism in taurine transporter or modulation of its function and/or expression by cell metabolic state or activation of transcription factors may affect the actual level of taurine being transported into the myofibers [134, 146, 152–154]. Hence caution should be taken when concluding about lack of taurine usefulness for human muscular system without an adequate control of all variables.

## Conclusion

We herein summarized the results obtained in about 30 years of research on taurine and skeletal muscle by us and other research groups. Taurine is far from themes of fashion science or from immediate interest in innovative drug development by Pharma Companies. Nevertheless the reason for such a long interest is that taurine acquired over the years a special appeal for its puzzling and multiple effects. We underlined the ability of taurine to control the function of ion channels and consequently membrane excitability as well as calcium homeostasis and excitation–contraction coupling. It has been highlighted that novel evidences are emerging regarding taurine mechanism of action, ranging from modulation of muscle metabolism to control of gene transcription, as well as in the specie-specific mechanisms underlying its intracellular levels in both chronic and acute conditions. These make the research on the topic “taurine and skeletal muscle” a continuous source of novel and exciting results allowing to renew the enthusiasm and novel working hypotheses. The wide and interconnected effects observed support a key role of the amino acid to ensure a proper muscle function and reinforce its interest as therapeutic agent in various inherited and acquired muscular disorders. The available evidences favor a greater effect of taurine in diseased condition accompanied by alterations in taurine concentration in muscle; similar benefit can occur in conditions where fluctuation in taurine level take place such as exercise, protein content in diet or post-natal development. Both acute and chronic effects of taurine supplementation are feasible, and likely occur with different time-scale although similarly interesting and important. Although a careful distinction has not been made, it is predictable that acute effects of taurine are better appreciable in situations of rapid fluctuations such as exercise, or when involving direct modulation of ion channel, or on muscles that are more dependable of external taurine such as fast-twitch ones. In parallel, chronic taurine effects, likely accompanied by changes in intracellular content, could be of value for long term control of neuromuscular function in progressive conditions, such as muscular dystrophy and disuse or aging-related dysfunction. At this regard more evidences are necessary to better understand the interest of taurine for ensuring a proper muscle function in human other than in animals. Consequently, a more clinically-oriented research will help to support the interest of taurine as novel and safer therapeutic approach of rare inherited muscle diseases and other myopathic states.
